# Pathway crosstalk perturbation network modeling for identification of connectivity changes induced by diabetic neuropathy and pioglitazone

**DOI:** 10.1186/s12918-018-0674-7

**Published:** 2019-01-07

**Authors:** Guillermo de Anda-Jáuregui, Kai Guo, Brett A. McGregor, Eva L. Feldman, Junguk Hur

**Affiliations:** 1 Department of Biomedical Sciences, University of North Dakota School of Medicine and Health Sciences, Grand Forks, North Dakota 58202 USA; 2 Department of Neurology, University of Michigan School of Medicine, Ann Arbor, MI 48109 USA; 3 Present address: Computational Genomics Division, Instituto Nacional de Medicina Genómica, 14610 Ciudad de México, Ciudad de México Mexico

**Keywords:** Pathway, Pathway network, Crosstalk, Perturbation analysis

## Abstract

**Background:**

Aggregation of high-throughput biological data using pathway-based approaches is useful to associate molecular results to functional features related to the studied phenomenon. Biological pathways communicate with one another through the crosstalk phenomenon, forming large networks of interacting processes.

**Results:**

In this work, we present the pathway crosstalk perturbation network (PXPN) model, a novel model used to analyze and integrate pathway perturbation data based on graph theory. With this model, the changes in activity and communication between pathways observed in transitions between physiological states are represented as networks. The model presented here is agnostic to the type of biological data and pathway definition used and can be implemented to analyze any type of high-throughput perturbation experiments. We present a case study in which we use our proposed model to analyze a gene expression dataset derived from experiments in a BKS-db/db mouse model of type 2 diabetes mellitus–associated neuropathy (DN) and the effects of the drug pioglitazone in this condition. The networks generated describe the profile of pathway perturbation involved in the transitions between the healthy and the pathological state and the pharmacologically treated pathology. We identify changes in the connectivity of perturbed pathways associated to each biological transition, such as rewiring between extracellular matrix, neuronal system, and G-protein coupled receptor signaling pathways.

**Conclusion:**

The PXPN model is a novel, flexible method used to integrate high-throughput data derived from perturbation experiments; it is agnostic to the type of data and enrichment function used, and it is applicable to a wide range of biological phenomena of interest.

**Electronic supplementary material:**

The online version of this article (10.1186/s12918-018-0674-7) contains supplementary material, which is available to authorized users.

## Introduction

The systems biology framework is useful for integrating large-scale data, such as those obtained from high-throughput genomic technologies. Pathway-based approaches can aggregate the results of these technologies regarding biological features of interest, which, if done correctly, can help interpret the phenomenological significance of the molecular observations at a functional level [1].

Network models are useful because they provide a generalized mathematical framework to describe biological states [2]. In this context, it is important to note that pathways themselves can be represented as networks, as pathways are sets of molecules with sequential interactions that lead to the activation or repression of effector molecules, leading to a biological function [3]. More importantly, given that pathways are not isolated, but in fact communicate with each other, these pathways form large networks that cover the range of biological functions associated with the functioning of a biological system [4].

Pathway crosstalk describes communication between functional pathways [5]. This concept is widely used in many biological settings to describe instances in which two functional pathways interact with each other; however, different researchers have used different interpretations of the concept [6]. The sequential molecular interactions in a pathway involve a flow of information (for instance, external stimuli through signaling transduction pathways). Since biomolecules may have more than one role and may be involved in more than one biological function, there may be interactions between these pathways. Crosstalk between pathways allows for alternative information flows between biological functions. This phenomenon provides the biological system with emergent properties such as robustness and adaptability to external perturbations, with biomedical implications [7].

The insights that can be obtained from network approaches to biology can be used to identify new leads in the study of complex diseases [8]. An example of such a complex disease is diabetes mellitus (DM). The most prevalent of diabetes’ chronic complications is diabetic neuropathy [9]. Although the exact mechanisms that lead to this condition in the diabetic patient are not completely described, growing evidence suggests that alterations in biological pathways may play an important role in the condition [10]. Currently, therapeutic options for this condition are limited [11]. Recent works [12] have focused on the role of lipid metabolism in the development of neuropathy and the use of pharmacological agents that target lipid metabolism, such as pioglitazone, a peroxisome proliferator-activated receptor gamma (PPARG) agonist with well-described antidiabetic effects [13].

This article presents a general method for constructing networks describing altered pathways between physiological states and perturbations in communication between these pathways. This method was used to construct networks to identify perturbations observed between the physiological, pathological, and pharmacological states in a murine model of type 2 DM (T2DM) peripheral neuropathy and the effects of treatment with pioglitazone. These networks provide insights into the importance of certain functional pathways in the different transitions between these states, which may in turn be used to drive novel experimental research on alternative pharmacological treatments for diabetic neuropathy.

## Methods

### The pathway Crosstalk perturbation network model

**Fig. 1:**
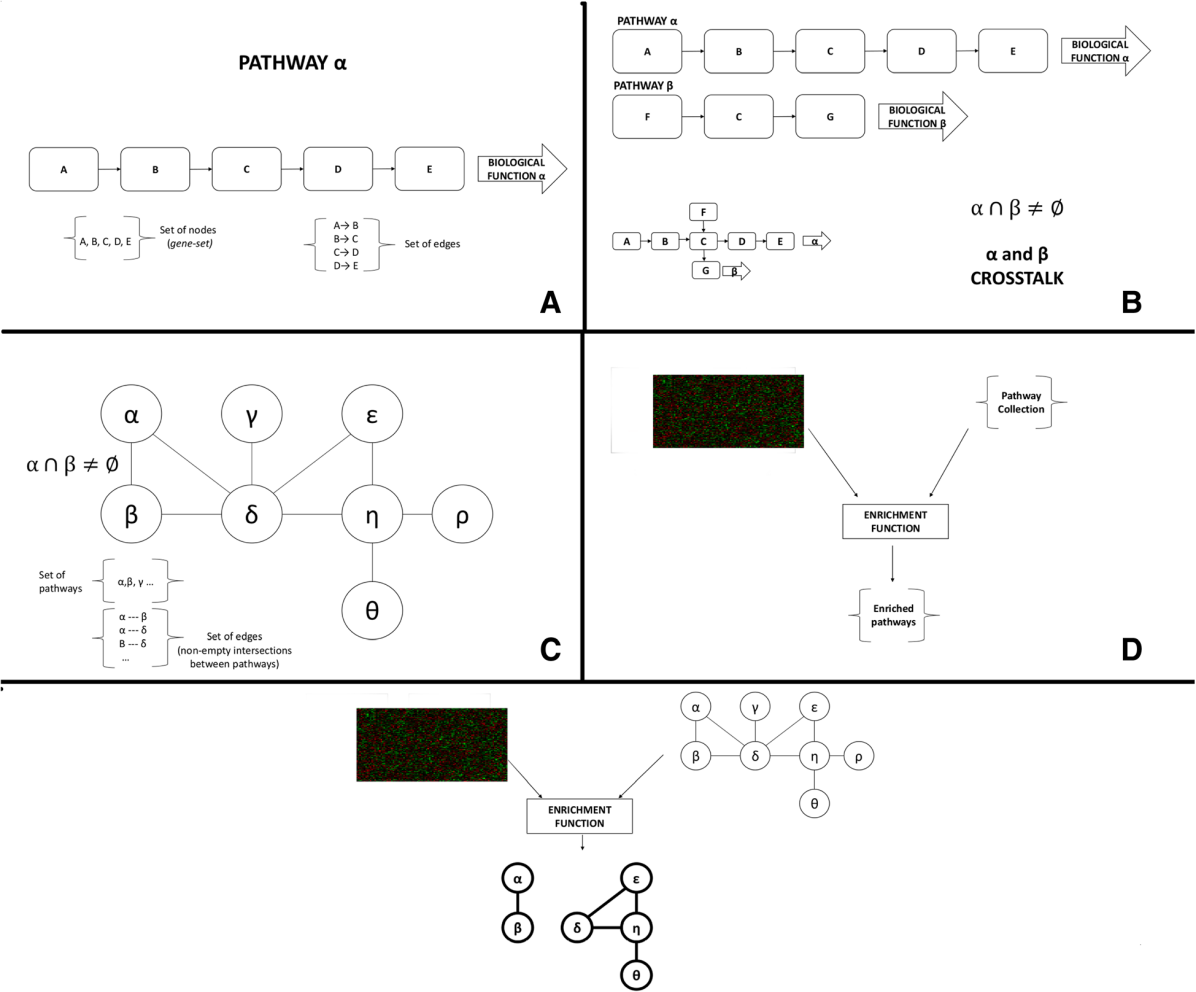
General representation of the pathway crosstalk perturbation network (PXPN) model. Panel **a** shows a pathway, a directed graph composed of nodes representing biomolecules and edges representing interactions between them that lead to a biological function. The set of nodes in a pathway is analogous to a gene set. Panel **b** shows an example of a pathway crosstalk in which two pathways that are involved in different biological functions share a molecule. Panel **c** shows a pathway crosstalk network (PXN) containing all pathways in a pathway collection and the links between pathways that crosstalk with one another (that is, pathways that have a nonempty intersection). Panel **d** shows a schematic representation of an enrichment analysis in which data from two different biological states and a list of pathways are taken by an enrichment function, which returns a list of pathways considered to be enriched. Finally, panel E shows the PXPN model in which an enrichment function takes data from two biological states and the set of nodes and edges that define a PXN and returns a network composed by enriched pathways and enriched crosstalk regions

In this work, the *pathway crosstalk perturbation network (PXPN)* is proposed as a model for integrating high-throughput perturbation biological data to gain insights into the changes in communication between functional biological processes. Figure 1 illustrates a schematic representation of the elements in the model, while the formal definitions of the elements in the PXPN model are provided in Additional file 1. The model is agnostic to the type of high-throughput data, the pathway description, and the statistical measure or algorithm used to define enrichment.

Basically, the PXPN model consists of four steps: 1) identification of perturbed pathways between two physiological states, 2) identification of crosstalk among the perturbed pathways, 3) identification of perturbations in the crosstalk regions between perturbed pathways, and 4) Network integration. A pseudocode representation of this model is available in Additional file 2. The scripts used in the current study are available in our Github repository (https://www.github.com/hurlab/pxpn_neuropathy).

Step 1 involves taking i) an expression dataset with information on two physiological conditions and ii) a list of pathways, defined by a single set of inclusion criteria and curatorial rules, such as those obtained from the same database. These are used as input for an enrichment function to obtain a list of pathways that are considered to be “enriched,” which in this context indicates a perturbation in pathway activity between the physiological states.

Step 2 involves looking for crosstalk between the pathways that were identified as perturbed in the previous step. Crosstalk between pathways is found if the pathways share genes, or, in other words, if the lists of genes for two pathways overlap (see definition in Additional file 1). The intersection between these two lists represents the genes that belong to the *crosstalk region (or regions)* between these pathways. Importantly, our research focused only on the crosstalk regions of pathways that were identified as perturbed in step 1.

Step 3 involves taking the list of crosstalk regions previously identified and using the expression data and the same enrichment function on it. Doing this allows identifying which crosstalk regions are perturbed themselves. Perturbation in the crosstalk region between two pathways indicates a change of activity in the molecules that are shared between pathways observed between the two physiological conditions, which in turn indicates a perturbation in the communication between pathways, as the expression of genes that connect the two pathways is being collectively perturbed.

Step 4 involves integrating the outputs of steps 1 and 3 into a network model. This is done by representing the perturbed pathways from step 1 as nodes in a graph, then establishing undirected links between pairs of pathways if the crosstalk region between them was identified as perturbed in step 3. The resulting undirected graph is referred to as a Pathway Crosstalk Perturbation Network, which represents the pathways that are perturbed between two biological states, along with the perturbations found in their crosstalk regions. This network model can be further analyzed from a graph-theoretic perspective.

The present research focused on the changes in pathway perturbation and communication through the analysis of changes in topology, modular structure, and connectivity, in PXPNs associated to physiological transitions. Given two phenotypes, such that one can give way to the other sequentially, the transition from the first to the second phenotype may involve the perturbation of a set of biological functions, which can be modeled using a PXPN. The progression from a physiologically healthy state to a pathological state of disease is an important biomedical case. This pathological state may, in turn, by using pharmacological agents, advance to a state of partially restored functionality. Hypothetically, a “perfect” drug could induce a final transition from the pharmacological state of partial functionality back to a healthy state indiscernible from the original physiological state. Each of these transitions can be modeled as three different PXPNs that represent the perturbations associated with each transition. As a case study, this model was implemented with data from a study of the effects of pioglitazone on murine T2DM-associated neuropathy.

### RNA-Seq data

RNA-Seq raw data were obtained from a previous study on pioglitazone’s effects on diabetic complications [12] using leptin receptor-deficient *db*/*db* mice, a model of T2DM. In brief, male C57BLKS (BKS) *db*/+ and *db*/*db* mice (BKS.Cg-m+/+Leprdb/J) were purchased from the Jackson Laboratory (Bar Harbor, ME). Mice were fed with or without 15 mg/kg pioglitazone (112.5 mg pioglitazone/kg chow for a dose of 15 mg/kg to the mouse) for 11 weeks starting from 5 weeks of age. Pioglitazone treatment normalized renal function and improved small nerve functions but did not improve large fiber functions. Four complication-prone tissues—sciatic nerve (SCN), dorsal root ganglia (DRG), glomeruli, and kidney cortex—were collected at 16 weeks of age and examined for their genome-wide gene expression profiles using RNA-Seq (HiSeq 2000 paired-end read length of 100 bases). The current study focused on the three groups of SCN data, including *db*/+ (nondiabetic denoted as “healthy” group), *db*/*db* (diabetic denoted as “disease” group), and *db*/*db* + Pio (diabetic with pioglitazone treatment denoted as “treatment” group). There were *n* = 6 samples in each group.

The raw sequencing reads were first cleaned by removing reads containing the adapter or poly-N and removing low-quality (quality score < 30) reads using Trimmomatic [14]. FastQC version 0.10.1 [15] was used to assess the quality of raw reads. Clean reads were mapped to the mouse reference genome GRCm38 (mm10) using Hisat2 [16]. The mapping summaries—such as the percentage of reads that were uniquely mapped, multiple mapped, or unmapped—were then collected from the log files of Hisat2 runs. FeatureCounts [17] was used to count reads mapped to individual genes. Only uniquely mapped reads were used in the counting step. Then, the counting metrics were collected from the summary file of each FeatureCounts run. Genes were omitted with zero expression across all samples for the correlation and differential expression analysis. Fragments per kilobase of exon per million mapped reads (FPKM) as a measurement of transcript expression were calculated using an in-house script.

### Pathway enrichment algorithm

The Reactome [18] collection of pathways was used in this study. We used the complete set of Reactome murine pathways available through the *Graphite* R/Bioconductor package [19]. The generally applicable gene set enrichment (GAGE) [20], a cutoff-free enrichment algorithm, was used to identify significantly enriched pathways that were perturbed by diabetes or treatment. The algorithm was run considering undirected perturbations, with an enrichment significance threshold set to q-value < 0.05.

### Network analysis

Calculations for topological parameters—degree, clustering coefficient (CC), network density, average path length, and number of connected components (islands in the network)—were carried out using Igraph [21] for R, NetworkX [22] for Python, and Cytoscape 3.3.0 [23]. Additionally, communities (subsets of nodes with high intraconnectivity and low outbound connections) were detected using the Infomap algorithm [24], as implemented in the Igraph package.

### Implementation for the study of physiological transitions in the murine diabetic neuropathy model

For this study of murine diabetic neuropathy, the previously described RNA-Seq expression dataset and pathways from the Reactome database were the inputs of the model. The differences between these groups represent the transitions observed in a patient. First, the patient transitions from a physiologically functional state to a pathological state (health to disease, denoted as *HTD*). Given therapy, the patient transitions from the pathological state to a pharmacologically modulated state (disease to treatment, denoted as *DTT*). Finally, with successful therapy the patient transitions back to the physiological state (treatment to health, denoted as *TTH*). Three networks, each representing one of these physiological transitions, were constructed. It is proposed that changes in pathway connectivity in different transitions indicate changes in the overall impact of a particular pathway activity in the observable phenotype.

### Null model

To evaluate the significance of the topological parameters of these three PXPNs, an ensemble of 5000 networks was generated for each transition using a null model by randomly rewiring the edges, with a rewiring probability proportional to the size of the intersection between two pathways (measured as the Jaccard index). For each network, each topological parameter was compared against the null model using a *Z*-test. This model allowed assessing whether particular topological properties of the obtained network differed from a randomly generated network containing the same number of nodes and edges (not all edges are possible, as not all pathways crosstalk with each other).

A second null model, for evaluating the overall capacity of the method of obtaining non-trivial network structures from gene expression measurements, was employed. For each of the three comparisons (*HTD*, *DTT*, and *TTH*), an ensemble of 1500 random expression datasets was generated by shuffling the gene labels of the original RNA-seq data. These data were used to generate PXPNs using the established pipeline and compared against those of the comparisons.

## Results

### Network overview

**Fig. 2:**
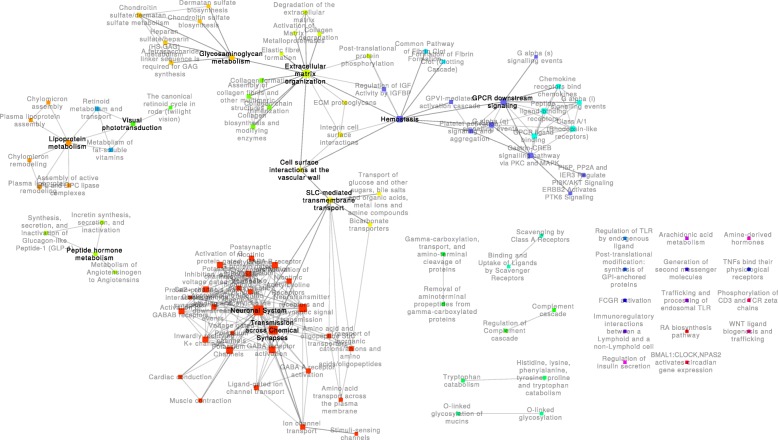
Health to disease (HTD) network. This network contains the 104 pathways that were perturbed in the transition from health to disease and the 222 crosstalk regions altered between these pathways. Communities of pathways related to similar biological functions are represented using different colors

**Fig. 3:**
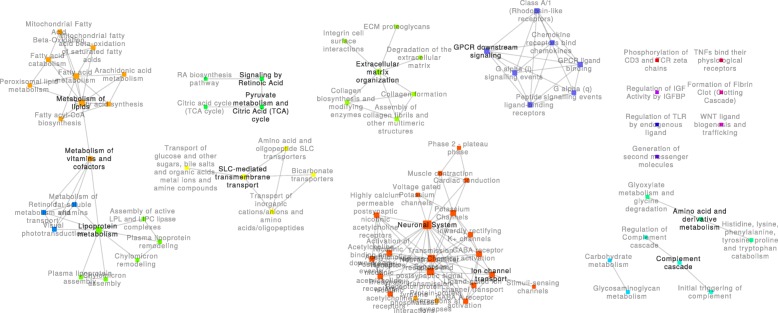
Disease to treatment (DTT) network. This network contains the 78 pathways that were perturbed in the transition from the disease state to a pharmacologically modulated state and the 149 crosstalk regions altered between these pathways. Communities of pathways related to similar biological functions are represented using different colors

**Fig. 4:**
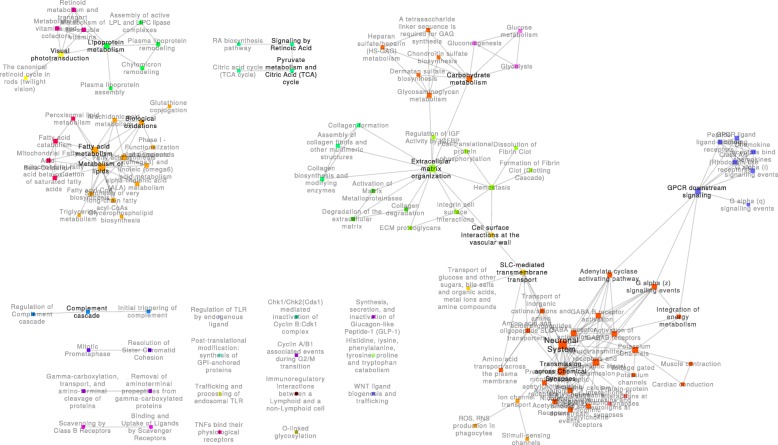
Treatment to health (HTD) network. This network contains the 110 pathways that were perturbed in the transition from health to disease and the 213 crosstalk regions altered between these pathways. Communities of pathways related to similar biological functions are represented using different colors

Using the proposed approach, each transition between physiological states was represented as a network with characteristic structural features. The generated networks may be found in Additional files 3, 4, and 5. Figure 2 illustrates the *HTD* network, which represents pathway alterations associated with the transition from the healthy state to the pathological neuropathic condition. Between these two states, 104 pathways were altered, and 222 significant alterations to the activity of crosstalk regions were observed. The second network, visualized in Fig. 3, represents the transition from the pathological state to a pharmacologically modulated state (the *DTT* network). This transition was associated with 78 altered pathways and 149 crosstalk perturbations among them. Finally, the *TTH* network, as illustrated in Fig. 4, describes the alterations found between the pharmacologically treated state and the healthy state, which would represent the perturbations observed in the transition back to the healthy state. This *TTH* network included 110 altered pathways, with 213 edges representing perturbed crosstalks between them. Additional file 6 illustrates the overlap among the pathways in these three networks; these pathways and their *q*-values for each transition are listed in Additional file 7.

Each physiological transition involves a precise combination of perturbed pathways, with specific communication patterns between them. This is evident in terms of similarity among the networks as each network has a portion of exclusive and shared nodes. In all three transitions, 53 pathways were perturbed and therefore represented as nodes in the network. The most similar networks in terms of both nodes and edges were the *DTT* and *TTH* networks (node Jaccard index = 0.55; edge Jaccard index = 0.45); the most different were the *HTD* and *TTH* networks (node Jaccard index = 0.49; edge Jaccard index = 0.43). Additional files 8 and 9 contain similar values among the three networks in terms of nodes and edges, respectively.

As each transition was associated with a specific network, each network was associated with specific structural characteristics. The communication between pathways associated with each transition between physiological states was different, resulting in a unique network topology. This in turn was reflected in network properties, such as the average path length, the clustering coefficient, and the distribution of nodes into connected components and communities inside connected components.

### Global network parameters

The PXPNs exhibited global topological properties that were nontrivial (Table 1). These properties were significantly different from those observed in the networks generated using the rewiring null model. The second null model, which constructed PXPNs from the original data with shuffled gene labels, showed that: 1) in most cases (1318, 1462, and 1190 out of 1500 random datasets for the *HTD*, *DTT*, and *TTH* comparisons, respectively), empty networks were generated, as no significant pathway perturbation was found; 2) networks with more than one edge included were generated in very few (106, 23, and 179, respectively) cases. Furthermore, these networks had trivial structures, such as stars and cliques. Therefore, the structural properties of PXPN networks may not be associated with random expression patterns, but may be associated with the underlying biological changes in pathway communication. Each PXPN had an associated degree distribution (Additional files 10, 11, 12), which was different from that of the random networks generated following the null model. Additional file 13 provides comparable parameters for the networks generated using the null model.

**Table 1:** Structural properties of pathway crosstalk perturbation networks for the transitions. *Specific density* refers to the number of perturbed crosstalk regions compared against the total number of possible crosstalks between the pathways in the network. HTD, health to disease; DTT, disease to treatment; TTH, treatment to health

	HTD	DTT	TTH
Nodes	104	78	110
Edges	222	149	213
Density	0.041	0.050	0.036
Specific density	222/1116	149/566	213/1051
Average degree	4.27	3.82	3.87
Clustering coefficient	0.579	0.620	0.513
Average shortest path	3.71	1.99	3.72
Connected components	22	16	19
Single nodes	14	7	11

The three experimentally derived networks had clustering coefficient values that were higher than the ones expected from the null model (for instance, the average clustering coefficient value for the *HTD* null model networks was 0.149). Nevertheless, the clustering coefficient values of all networks were comparable (ranging from 0.513 to 0.620). Average path lengths were slightly higher in the experimentally derived networks than in the null model networks in the cases of the *HTD* and *TTH* transitions. Interestingly, in the case of the *DTT* transition, the average path length was considerably lower than the one predicted by the null model (1.99, compared with the predicted 3.26). This result suggests that the transition induced by pharmacological treatment on the pathological state involves perturbations with short-range pathway communication, whereas the perturbations from and to the physiological state require longer-range changes in communication.

All three networks had low density for edges. Since not all edges were biologically possible, as not all pathways are able to crosstalk, comparing the number of edges against the total possible crosstalks between the pathways in each network is important; we refer to this as the *specific density* of a PXPN. In the *HTD* network, 19% of possible crosstalks were perturbed, where 26 and 20% of possible crosstalks were perturbed in the *DTT* and *TTH* networks, respectively. Derived from this is the observation of pathways that while they have a large crosstalk potential with other perturbed pathways in a transition, they appear disconnected nonetheless. For instance, in the *HTD* network, the “regulation of insulin secretion” could potentially connect to 32 pathways, but it was disconnected, indicating that the crosstalk of this pathway was not altered during the transition from health to disease. This lack of observed connectivity indicates that, in this transition, at least at the level of gene expression perturbation, this pathway has little system-wide influence.

### Connectivity and modular structure of networks

Our proposed model allowed the representation of the alterations between physiological states in pathway activity and communication as a graph. The structures of these networks were nontrivial and different from those of the random networks because the connections reflect the perturbation of crosstalk regions associated to each physiological transition. Therefore, there are differences with respect to the connected components (subgraphs in which any pair of nodes has a path between them) and communities (modules inside a connected component in which the nodes belonging to the same module have a higher number of edges between them than the nodes outside of the module). Differences in the organization of these networks are indicative of specific communications between biological processes that were altered in each physiological transition.

The *HTD* network (Fig. 2) was composed of 22 connected components, 14 of which were single nodes. The largest connected component contained 66 pathways (~ 63% of all 104 pathways), which were related to the “neuronal system” processes. This component also included other smaller communities related to “solute carrier (SLC)-mediated transmembrane transport,” “extracellular matrix (ECM) organization,” “G-protein coupled receptor (GPCR) ligand binding,” and a community containing diverse pathways, such as “hemostasis” and “GPCR downstream signaling.” The second largest connected component contained 10 pathways (~ 10% of all nodes), which were mainly related to “lipoprotein metabolism.”

The *DTT* network (Fig. 3) was composed of 16 connected components, 7 of which were single nodes. In this network, the largest connected component contained only 21 pathways (~ 27% of all 78 pathways), which were similar to those found in the “neuronal system” community in the *HTD* network. The second largest connected component (19 pathways corresponding to ~ 24% of the network nodes) was composed of three communities: one comparable to the “lipoprotein metabolism” community in the *HTD* network, another related to the “metabolism of lipids,” and the other containing three pathways related to “retinoids.” Three other connected components were comparable to the communities found in the *HTD* network, such as “ECM organization” (8.97%), “GPCR ligand binding” (8.97%), and “SLC-mediated transmembrane transport” (6.41%). Another connected component with four pathways related to the TCA cycle was also found.

The *TTH* network (Fig. 4) was composed of 19 connected components, 11 of which were single nodes. This network was dominated by the largest connected component, which contained 59 pathways (~ 54% of all 110 pathways). The communities in this component were similar to those in the largest component of the *HTD* network, including the “neuronal system,” “SLC-mediated transmembrane transport,” “ECM organization,” “GPCR-ligand binding,” and “GPCR-downstream signaling.” Interestingly, a new community emerged, containing the “glycolysis,” “gluconeogenesis,” and “glucose metabolism” pathways, which were connected to the “ECM organization” community. A notable difference between this network and the *HTD* network was the changes in the communication of the GPCR community, which became disconnected from the “ECM organization” community and connected to the “neuronal” community.

## Discussion

Biological systems function through the integration of different molecular processes. Pathway crosstalk occurs because biomolecules are involved in more than one single biological function. This work presents the PXPN model, a graph theory approach for analyzing high-throughput gene expression perturbation data. Using the PXPN model, large-scale data representing the differences between physiological states can be aggregated into network structures that not only have reduced dimensionality, but also have functional significance: they describe known biological processes. With this approach, representing the inherently dynamic nature of physiological transitions as networks is possible; the resulting networks can be analyzed with a myriad of tools derived from graph and complex network theory.

Much previous research on pathway crosstalk focused on the phenomenon with the intention of reducing the number of identified functions from pathway enrichment analysis [25–27]. The goal of generating an integrated network representation of pathway communication is currently being explored through many different perspectives [28, 29]. Our PXPN model assumes that biological perturbations lead to changes in both pathway activity and the communication of these pathways through crosstalk. Therefore, pathway perturbation analysis may be approached through a global representation of the phenomenon, such as a network. By generating a network that integrates information on pathway and crosstalk perturbation, the perturbation phenomenon may be studied using tools derived from graph theory, allowing us to have individual and global descriptors of the phenomenon in terms of topological properties. Different approaches may be complementary, and their use would depend on the individual research questions to be answered.

In this work, the PXPN model was used to gain a topological description of the contribution of pathways to the network. The focus was on the modular structure of each PXPN, both at the level of connected components and at the level of communities inside the connected components, as well as how the emergence and loss of crosstalk perturbation led to an evolution of the modular structure as the system transitioned. The PXPN model on the case study of murine diabetic neuropathy was used to identify certain functional pathways that appear to be important to the progression of phenotypes, based on their network properties. Also described was how crosstalk activity between these pathways changes through physiological transitions and how this affects the organization of the network of pathways.

In this work, bioenergetic pathways related to glucose, lipid, and TCA cycle metabolism were scattered over multiple disconnected components disconnected from one another. The role of bioenergetic pathways in the development of diabetic neuropathy has been an active area of recent research [30–33]. Our observations suggest an independent contribution of these pathways to the transitions between physiological states. Interestingly, only the metabolism of lipoproteins was altered in the transition from health to disease (the *HTD* network; Fig. 2); TCA cycle metabolism and general lipid metabolism emerged only with the treatment of pioglitazone (the *DTT* network; Fig. 3). Carbohydrate metabolism pathways, including glycolysis and gluconeogenesis pathways, were only associated with the transition from the pharmacological to the health state (the *HTD* network; Fig. 4) and were the only instance of bioenergetic pathways belonging to the largest connected component of any network.

Changes to the composition and function of the ECM pathways have been reported to play a significant role in the loss of nerve fibers during the progression of diabetic neuropathy [34]. In each of the three experimental PXPNs, ECM pathways were found to be organized in distinctive communities. In the transitions from and to the health state, crosstalk between the ECM- and neuronal system-related pathways was enriched, forming a large connected component. In both cases, this crosstalk was indirect through the SLC-mediated transmembrane transport pathway; the smaller size of the largest component in the *DTT* network can be partially explained by a lack of enriched crosstalk through the SLC-mediated transmembrane transport pathway. As previously mentioned, glucose bioenergetic metabolism pathways were only found in the *TTH* network, crosstalking with the ECM pathways and the glycosaminoglycan (GAG) metabolism pathways. GAGs are known to play critical roles in the development of the central nervous system [35] and are involved in the processes of axon regeneration in the peripheral nervous system [36].

GPCR signaling is widely known to be an important mechanism of signal transduction and has been highly studied in the biomedical setting as a drug target [37]. In this study, changes in the connectivity of GPCR signaling pathways were identified. In the context of the *HTD* transition, GPCR signaling was directly connected to the largest connected component of the network through crosstalk with the hemostasis pathway. Hemostasis perturbation was lost in the *DTT* transition, leading to the formation of an isolated component of GPCR signaling. Finally, while hemostasis perturbation was found again in the *TTH* transition, the crosstalk between this pathway and GPCR was lost, while new connections from the GPCR pathways to the neuronal system community emerged through three pathways: “G alpha (z) signaling events,” “integration of energy metabolism,” and “adenylate cyclase–activating pathway.” Recently, the parabrachial pituitary adenylate cyclase–activating polypeptide (PACAP) was shown to have an increased expression in chronic pain [38]; our results indicate that the “adenylate cyclase–activating pathway” shows a perturbation in the transition to heath, which may connect neuronal function pathways and GPCR signaling pathways.

**Fig. 5:**
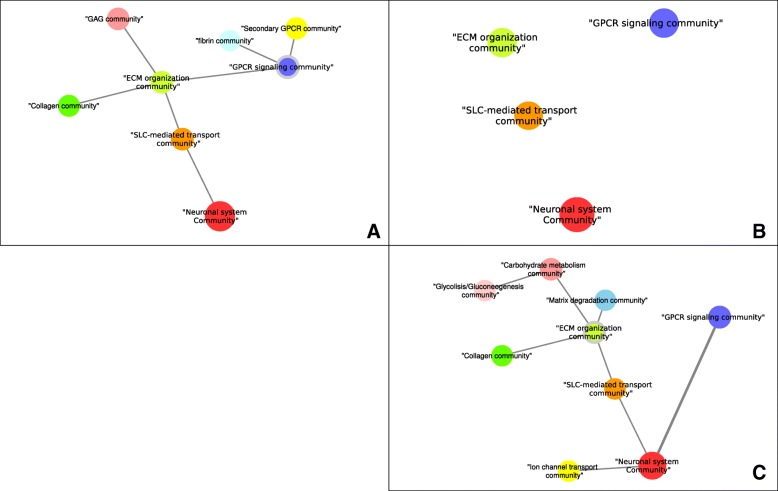
Pathway rewiring associated to different biological transitions. Each panel shows a network of communities identified in each transition: health to disease in panel **a**, disease to treatment in panel **b**, and treatment to health in panel **c**. In panel **a**, the “GPCR signaling community” is connected to the “ECM organization community” but not to the “neuronal system community.” In panel **b**, only four communities—“ECM organization community,” “GPCR signaling community,” “SLC-mediated transport community,” and “neuronal system community”—survived with no connections between communities. In panel **c**, the “GPCR signaling community” exhibits a rewiring, with a new connection (shown with a thicker line in the image) to the “neuronal system community.” A gray outline indicates the community that contains the “hemostasis” pathway, which changes in each transition as a consequence of pathway rewiring: it initially belongs to the “GPCR signaling community,” it is not found in any community in panel **b** (as it is not perturbed in this transition), and finally, it belongs to the “ECM organization community”

Figure 5 summarizes the rewiring observed between the communities associated with the ECM, GPCR, and neuronal system pathways, showing a “network of networks” of communities in each biological transition. Our PXPN model identifies changes in communication between pathways, which can be associated with the progression between altered states. With it, identifying which crosstalking pathways are more relevant to each biological transition is possible, which may in turn guide new experimental research.

## Conclusion

In this work, we presented a model to represent the alterations in pathway activity and communication between physiological states of clinical importance. This PXPN model represents each physiological transition as a network of perturbed and interacting pathways with a unique nonrandom structure. These networks reflect the changes in the functional biological processes that are observed in the transitions between different physiological states. More importantly, these networks give insights into the importance that the communication between biological functions may have in the progression between physiological states.

The PXPN model is agnostic to the type of perturbation dataset and can be applied to analyze a variety of experimental settings; when given an expression/perturbation dataset and a suitable enrichment method, it is possible to generate a PXPN. The topological properties of this network, as well as the biological insights that can be unveiled from its analysis, depend on the nature of the data and the assumptions of the enrichment methodology. In this work, a diabetic neuropathy animal model (development of diabetic neuropathy in *db/db* mice and treatment of pioglitazone) was used as a case study. Our model identified changes in pathway connectivity, such as the rewiring between pathways of extracellular matrix, neuronal system, and GPCR signaling, in different biological transitions of clinical importance.

## Additional files

Additional file 1: Model definitions. (DOCX 13 kb)

Additional file 2: Text file with the PXPN model pseudocode. (DOCX 16 kb)

Additional file 3: Health to Disease Network in GML format. (GML 42 kb)

Additional file 4: Disease to Treatment Network in GML format. (GML 30 kb)

Additional file 5: Treatment to Health Network in GML format. (GML 42 kb)

Additional file 6: Venn diagram of pathways in PXPNs in TIFF format. (TIFF 413 kb)

Additional file 7: Pathway enrichment (q-values) for each transition, in XLSX format. (XLSX 13 kb)

Additional file 8: Jaccard index matrix of similarity for PXPN nodes in XLSX format. (XLSX 9 kb)

Additional file 9: Jaccard index matrix of similarity for PXPN edges in XLSX format. (XLSX 9 kb)

Additional file 10: Degree distribution for the health to disease (*HTD*) network (solid, black) and corresponding null model distributions (colored outlines); PDF format. (PDF 10 kb)

Additional file 11: Degree distribution for the disease to treatment (*DTT*) network (solid, black) and corresponding null model distributions (colored outlines); PDF format. (PDF 8 kb)

Additional file 12: Degree distribution for the treatment to health (*TTH*) network (solid, black) and corresponding null model distributions (colored outlines); PDF format. (PDF 9 kb)

Additional file 13: Parameters of the random networks generated with the null model in XLSX format. (XLSX 21 kb)

## Abbreviations

CC: Clustering coefficient
DM: Diabetes mellitus
DTT: Disease to treatment
ECM: Extracellular matrix
GAGE: Generally applicable geneset enrichment
GPCR: G-protein coupled receptor
HTD: Health to disease
PPARG: Peroxisome proliferator-activated receptor gamma
PXPN: Pathway crosstalk perturbation network
SLC: Solute carrier
T2DM: Type 2 diabetes mellitus
TTH: Treatment to health

## References

[1] Garcia-Campos MA, Espinal-Enriquez J, Hernandez-Lemus E (2015). Pathway analysis: state of the art. Front Physiol.

[2] Barabasi AL, Oltvai ZN. Network biology: understanding the cell's functional organization. Nat Rev Genet. 2004;5(2):101–113. PMID: 14735121 10.1038/nrg1272 14735121

[3] Bhalla US, Iyengar R. Emergent properties of networks of biological signaling pathways. Science. 1999;283(5400):381–387. PMID: 9888852. 10.1126/science.283.5400.381 9888852

[4] De Anda-Jáuregui G, Mejía-Pedroza RA, Espinal-Enríquez J, Hernández-Lemus E (2015). Crosstalk events in the estrogen signaling pathway may affect tamoxifen efficacy in breast cancer molecular subtypes. Comput Biol Chem.

[5] Gilbert SF. Cross-talk between pathways. Dev Biol. 2000.

[6] Vert G, Chory J. Crosstalk in Cellular Signaling: background noise or the real thing? Dev Cell. 2011:1179. 10.1016/j.devcel.2011.11.006 PMC3281494 22172668

[7] Sun X, Bao J, You Z, Chen X, Cui J (2016). Modeling of signaling crosstalk-mediated drug resistance and its implications on drug combination. Oncotarget.

[8] Barabási A-L, Gulbahce N, Loscalzo J (2011). Network medicine: a network-based approach to human disease. Nat Rev Genet.

[9] Pop-Busui R, Boulton AJM, Feldman EL, Bril V, Freeman R, Malik RA (2017). Diabetic neuropathy: a position statement by the American diabetes association. Diabetes Care.

[10] Feldman EL, Nave KA, Jensen TS, Bennett DLH. New Horizons in Diabetic Neuropathy: Mechanisms, Bioenergetics, and Pain. Neuron. 2017;93(6):1296–1313. PMID: 28334605. 10.1016/j.neuron.2017.02.005 PMC5400015 28334605

[11] Waldfogel JM, Nesbit SA, Dy SM, Sharma R, Zhang A, Wilson LM, Bennett WL, Yeh HC, Chelladurai Y, Feldman D et al. Pharmacotherapy for diabetic peripheral neuropathy pain and quality of life: A systematic review. Neurology. 2017;88(20):1958–1967. PMID: 28341643. 10.1212/WNL.0000000000003882 28341643

[12] Hinder LM, Park M, Rumora AE, Hur J, Eichinger F, Pennathur S (2017). Comparative RNA-Seq transcriptome analyses reveal distinct metabolic pathways in diabetic nerve and kidney disease. J Cell Mol Med.

[13] Yasmin S, Jayaprakash V: Thiazolidinediones and PPAR orchestra as antidiabetic agents: From past to present. Eur J Med Chem. 2017;126:879–893. PMID: 27988463. 10.1016/j.ejmech.2016.12.020 27988463

[14] Bolger AM, Lohse M, Usadel B (2014). Trimmomatic: a flexible trimmer for Illumina sequence data. Bioinformatics.

[15] Andrews S. FastQC: A quality control tool for high throughput sequence data. 2010. http://www.bioinformatics.babraham.ac.uk/projects/fastqc/.

[16] Sirén J, Välimäki N, Mäkinen V (2014). HISAT2 - fast and sensitive alignment against general human population. IEEE/ACM Trans Comput Biol Bioinforma.

[17] Liao Y, Smyth GK, Shi W (2014). FeatureCounts: an efficient general purpose program for assigning sequence reads to genomic features. Bioinformatics.

[18] Fabregat A, Sidiropoulos K, Garapati P, Gillespie M, Hausmann K, Haw R (2016). The reactome pathway knowledgebase. Nucleic Acids Res.

[19] Sales G, Calura E, Cavalieri D, Romualdi C (2012). Graphite - a Bioconductor package to convert pathway topology to gene network. BMC Bioinformatics..

[20] Luo W, Friedman MS, Shedden K, Hankenson KD, Woolf PJ (2009). GAGE: generally applicable gene set enrichment for pathway analysis. BMC Bioinformatics..

[21] Csardi G, Nepusz T. The igraph software package for complex network research. InterJournal. 2006;(Complex Sy):1695.

[22] Hagberg AA, Schult DA, Swart PJ. “Exploring network structure, dynamics, and function using NetworkX”. In Proceedings of the 7th Python in Science Conference (SciPy2008), Gäel Varoquaux, Travis Vaught, and Jarrod Millman (Eds), (Pasadena, CA USA). 2008. pp. 11–15.

[23] Su G, Morris JH, Demchak B, Bader GD: Biological network exploration with Cytoscape 3. Curr Protoc Bioinformatics. 2014;47:8 13 11–24. PMID: 25199793. 10.1002/0471250953.bi0813s47 PMC4174321 25199793

[24] Rosvall M, Axelsson D, Bergstrom CT (2009). The map equation. Eur Phys J Spec Top.

[25] Tomoiaga A, Westfall P, Donato M, Draghici S, Hassan S, Romero R (2016). Pathway crosstalk effects: shrinkage and disentanglement using a Bayesian hierarchical model. Stat Biosci.

[26] Ogris C, Guala D, Helleday T, Sonnhammer ELL (2017). A novel method for crosstalk analysis of biological networks: improving accuracy of pathway annotation. Nucleic Acids Res.

[27] Simillion C, Liechti R, Lischer HEL, Ioannidis V, Bruggmann R (2017). Avoiding the pitfalls of gene set enrichment analysis with SetRank. BMC Bioinformatics.

[28] Chen KM, Tan J, Way GP, Doing G, Hogan DA, Greene CS. PathCORE-T: identifying and visualizing globally co-occurring pathways in large transcriptomic compendia. BioData Min. 2018;11:14. PMID: 29988723. 10.1186/s13040-018-0175-7 PMC6029133 29988723

[29] Pita-Juarez Y, Altschuler G, Kariotis S, Wei W, Koler K, Green C (2018). The pathway Coexpression network: revealing pathway relationships. PLoS Comput Biol.

[30] Sas KM, Kayampilly P, Byun J, Nair V, Hinder LM, Hur J, Zhang H, Lin C, Qi NR, Michailidis G et al. Tissuespecific metabolic reprogramming drives nutrient flux in diabetic complications. JCI Insight. 2016;1(15):e86976. PMID: 27699244. 10.1172/jci.insight.86976 PMC5033761 27699244

[31] Hinder LM, Vivekanandan-Giri A, McLean LL, Pennathur S, Feldman EL (2013). Decreased glycolytic and tricarboxylic acid cycle intermediates coincide with peripheral nervous system oxidative stress in a murine model of type 2 diabetes. J Endocrinol.

[32] Hinder LM, O’Brien PD, Hayes JM, Backus C, Solway AP, Sims-Robinson C (2017). Dietary reversal of neuropathy in a murine model of prediabetes and metabolic syndrome. Dis Model Mech.

[33] Rumora AE, Lentz SI, Hinder LM, Jackson SW, Valesano A, Levinson GE, et al. Dyslipidemia impairs mitochondrial trafficking and function in sensory neurons. FASEB J. 2017. 10.1096/fj.201700206R PMC6191072 28904018

[34] Hill R. Extracellular matrix remodelling in human diabetic neuropathy. J Anat. 2009;214(2):219–225. PMID: 19207983. 10.1111/j.1469-7580.2008.01026.x PMC2667879 19207983

[35] Sugahara K, Mikami T, Uyama T, Mizuguchi S, Nomura K, Kitagawa H (2003). Recent advances in the structural biology of chondroitin sulfate and dermatan sulfate. Curr Opin Struct Biol.

[36] Groves ML, McKeon R, Werner E, Nagarsheth M, Meador W, English AW (2005). Axon regeneration in peripheral nerves is enhanced by proteoglycan degradation. Exp Neurol.

[37] Hauser AS, Attwood MM, Rask-Andersen M, Schioth HB, Gloriam DE. Trends in GPCR drug discovery: new agents, targets and indications. Nat Rev Drug Discov. 2017;16(12):829–842. PMID: 29075003. 10.1038/nrd.2017.178 PMC6882681 29075003

[38] Missig G, Mei L, Vizzard MA, Braas KM, Waschek JA, Ressler KJ (2017). Parabrachial pituitary adenylate cyclase-activating polypeptide activation of amygdala endosomal extracellular signal–regulated kinase Signaling regulates the emotional component of pain. Biol Psychiatry.

