# Correction to: Pathway crosstalk perturbation network modeling for identification of connectivity changes induced by diabetic neuropathy and pioglitazone

**DOI:** 10.1186/s12918-019-0707-x

**Published:** 2019-03-13

**Authors:** Guillermo de Anda-Jáuregui, Kai Guo, Brett A. McGregor, Eva L. Feldman, Junguk Hur

**Affiliations:** 1 Department of Biomedical Sciences, University of North Dakota School of Medicine and Health Sciences, Grand Forks, North Dakota 58202 USA; 2 Department of Neurology, University of Michigan School of Medicine, Ann Arbor, MI 48109 USA; 3 Present address: Computational Genomics Division, Instituto Nacional de Medicina Genómica, 14610 Ciudad de México, Mexico

**Correction to: BMC Systems Biology 20149 13:1**

**https://doi.org/10.1186/s12918-018-0674-7**

Following publication of the original article [1], it was highlighted that the funding statement was not correct. This Correction article shows the correct and incorrect Funding section.

Correct:

The National Institute of Diabetes and Digestive and Kidney Diseases (NIDDK; 1R24082841 to E.L.F. and J.H.), the NIDDK Diabetic Complications Consortium Pilot Grant (DiaComp, https://diacomp.org/; DK076169; sub-award #25034–75 to J.H.), and University of North Dakota Post-Doc Pilot Grant (to K.G.), and the North Dakota EPSCoR through National Science Foundation grant #OIA-1355466 (to B.A.M.). Funding bodies had no role in the design, execution, or analysis of this work.

Incorrect:

The National Institute of Diabetes and Digestive and Kidney Diseases (NIDDK; 1R24082841 to E.L.F. and J.H.), the NIDDK Diabetic Complications Consortium Pilot Grant (DiaComp, https://diacomp.org/; DK076169; sub-award #25034–75 to J.H.), and University of North Dakota Post-Doc Pilot Grant (to K.G.). Funding bodies had no role in the design, execution, or analysis of this work.
