# Supplementary material for: Pathway crosstalk perturbation network modeling for identification of connectivity changes induced by diabetic neuropathy and pioglitazone
Source: BMC Syst Biol. 2019 Jan 7;13:1. doi: 10.1186/s12918-018-0674-7 (PMC6322225; doi:10.1186/s12918-018-0674-7)
Supplement: Supplementary file 1 — Model definitions. (DOCX 13 kb) [file 12918_2018_674_MOESM1_ESM.docx]

# Model definitions

A *pathway* is defined as a directed graph *PW(V,E)*, containing a set *V* of nodes, representing a set of biomolecules, and a set *E* of edges, representing sequential interactions between such molecules, whose activity can be associated to a biologically relevant function. The biomolecules in *V* are generally proteins; however, as the technologies for genome-wide expression measurement are more developed and more generally used, gene transcripts are used as proxies. Hence, *V* can also be described as a gene set*.*

A *pathway collection* is a list of pathways, as defined and collected by a single set of inclusion criteria and curatorial rules. A *pathway crosstalk* between two pathways is defined if the intersection of the nodes in these two pathways is not empty. A *pathway crosstalk network* may be defined for any list of pathways, including a pathway collection, as a graph *G(PW,X)* containing a set of *PW*s as nodes representing *pathways* and a set *X* of edges representing the *crosstalk region* between pathways.

An enrichment function*,* ef*,* *is a function that, given a list of gene sets L and the state of biomolecules in two physiological conditions, S1 and S2, will return a subset of L containing significantly altered (perturbed) gene sets.* *Given a* G(PW,X)*, two physiological conditions, S1 and S2, and an enrichment function* ef*, a* PXPN(EPW, EX), *a subgraph of* G*, is defined such that* EPW *belongs to* ef(PW) *and* EX *belongs to* ef(X)*.*
