# Supplementary material for: Pathway crosstalk perturbation network modeling for identification of connectivity changes induced by diabetic neuropathy and pioglitazone
Source: BMC Syst Biol. 2019 Jan 7;13:1. doi: 10.1186/s12918-018-0674-7 (PMC6322225; doi:10.1186/s12918-018-0674-7)
Supplement: Supplementary file 2 — Text file with the PXPN model pseudocode. (DOCX 16 kb) [file 12918_2018_674_MOESM2_ESM.docx]

#Pathway Crosstalk Perturbation Network Model

#PXPN

#Pseudocode - R

#Step 1) Identification of pathway perturbation between physiological states

#Step 2) Identification of crosstalk between perturbed pathways

#Step 3) Identification of crosstalk perturbation between physiological states

#Step 4) Network integration

#further network analyses

##inputs

State_1_data #expression matrix,

#rows are genes

#columns are samples

#for physiological state 1

State_2_data #expression matrix,

#rows are genes

#columns are samples

#for physiological state 2

pathway_list #a list of pathways, set of genes associated to function

#Step 1) Identification of pathway perturbation between physiological states

enrichment_function <- function(State_1_data,

State_2_data,

pathway_list

#identifies which pathways are perturbed

#between conditions

)

#return a list of Perturbed pathways between conditions

Perturbed_Pathway_List <-enrichment_function(State_1_data,

State_2_data,

pathway_list

)

#Step 2) Identification of crosstalk between perturbed pathways

For(i in Perturbed_Pathway_List){

For(j in Perturbed_Pathway_List){

if(intersect(i,j)!= 0){

crosstalk = TRUE

crosstalk(i,j) = intersect(i,j) #the set of genes shared by i and j are

#the crosstalk region

#between pathways i and j

#add them to the crosstalk list

crosstalk_list <- add(crosstalk(i,j))

}else

crosstalk == FALSE #if intersect(i,j) is empty, i and j do not crosstalk

}

}

return(crosstalk_list)

crosstalk_list # a list of crosstalk regions between perturbed pathways

#Step 3) Identification of crosstalk perturbation between physiological states

perturbed_crosstalk = enrichment_function(State_1_data,

State_2_data,

crosstalk_list

)

perturbed_crosstalk #a list of crosstalk regions between perturbed pathways

#which show perturbation, as detected by the

#enrichment function

#Step 4) Network integration

nodes = Perturbed_Pathway_List #nodes are perturbed pathways

for(i in nodes){

for(j in nodes){

if(crosstalk(i,j)%in%perturbed_crosstalk){

edge(i,j) = TRUE #establish an undirected link

#between pathways i and j

#if the crosstalk region is perturbed

}else

edge(i,j) = FALSE #pathways whose crosstalk region is not perturbed

#have no link between them

}

}

network = graph(nodes, edges)

#further network analyses

##Topological characterization of the network

###Network density

###Degree distribution

###Network Diameter

###Clustering coefficient

###Average path length

##Individual node characterization

###Centrality measures

####Degree

####Betweenness

#Clustering coefficient

##Large-scale topology

###Component distribution

###Community/modular structure

##Network comparisons between phenotypes

###Preserved/Lost nodes and edges

###Community evolution

#etc...
