# Supplementary figures and images for: Pathway crosstalk perturbation network modeling for identification of connectivity changes induced by diabetic neuropathy and pioglitazone
Source: BMC Syst Biol. 2019 Jan 7;13:1. doi: 10.1186/s12918-018-0674-7 (PMC6322225; doi:10.1186/s12918-018-0674-7)

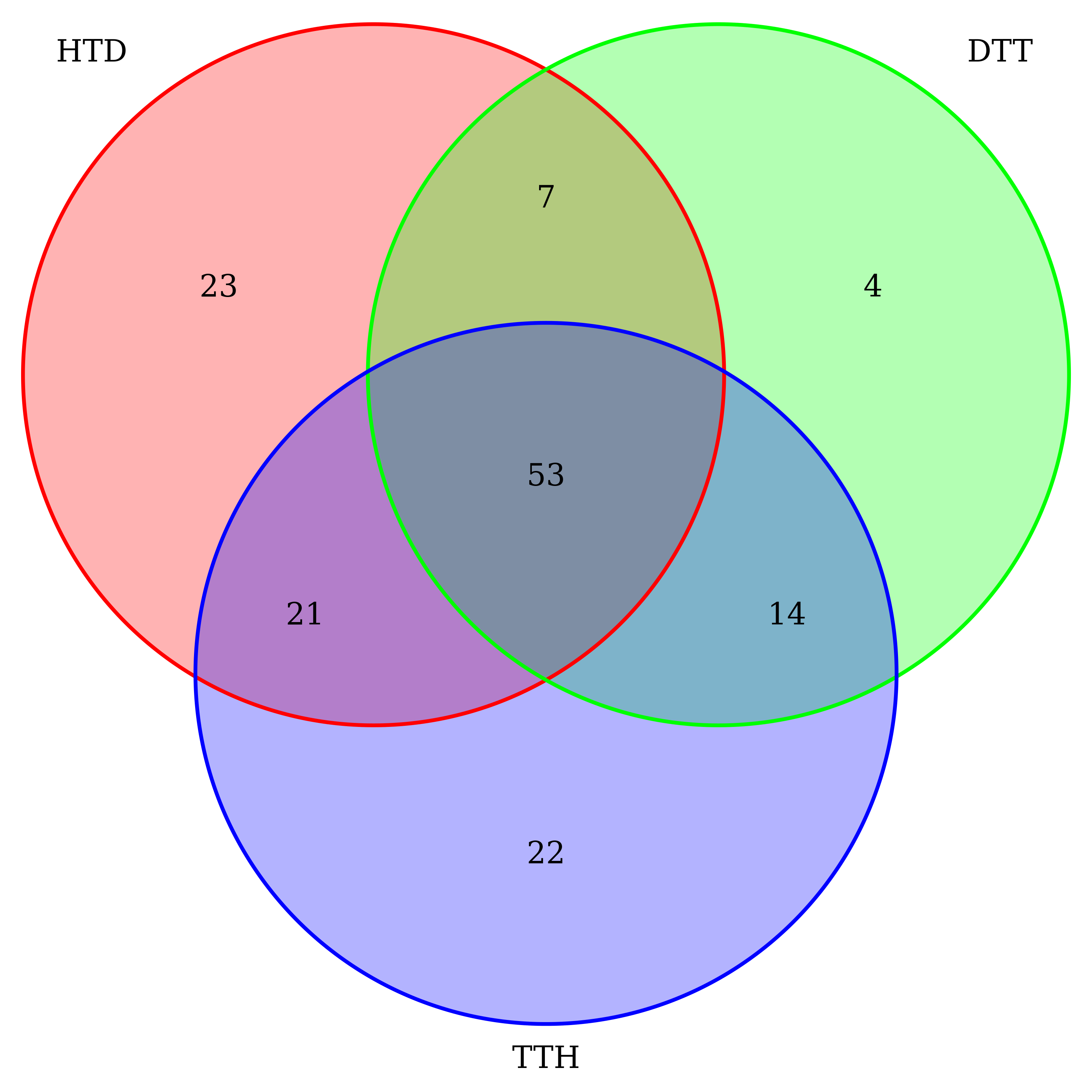

Supplement: Supplementary file 6 — Venn diagram of pathways in PXPNs in TIFF format. (TIFF 413 kb) [file 12918_2018_674_MOESM6_ESM.tiff]

Degree distribution

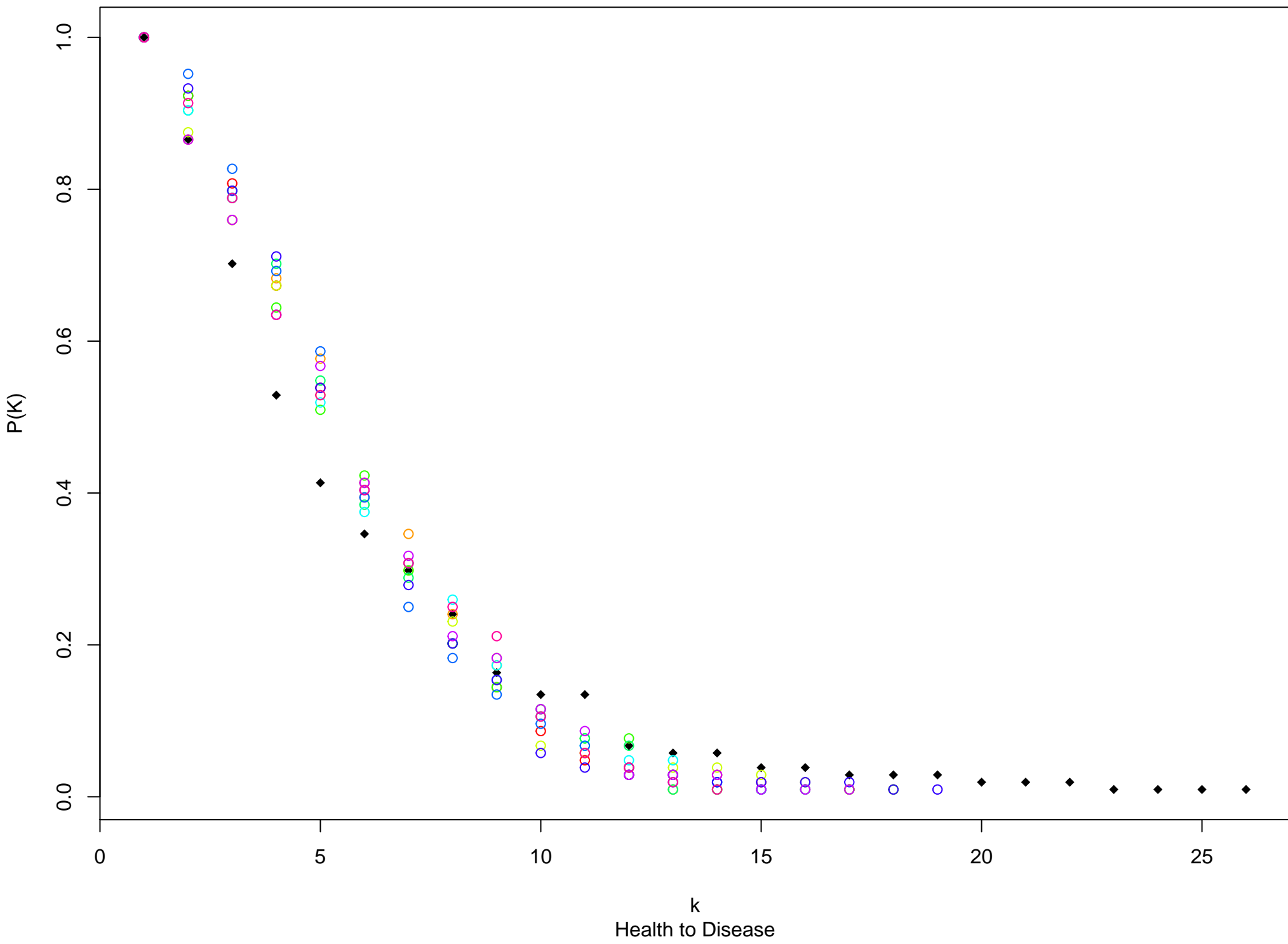

Supplement: Supplementary file 10 — Degree distribution for the health to disease (HTD) network (solid, black) and corresponding null model distributions (colored outlines); PDF format. (PDF 10 kb) [file 12918_2018_674_MOESM10_ESM.pdf]

## Disease to Treatment

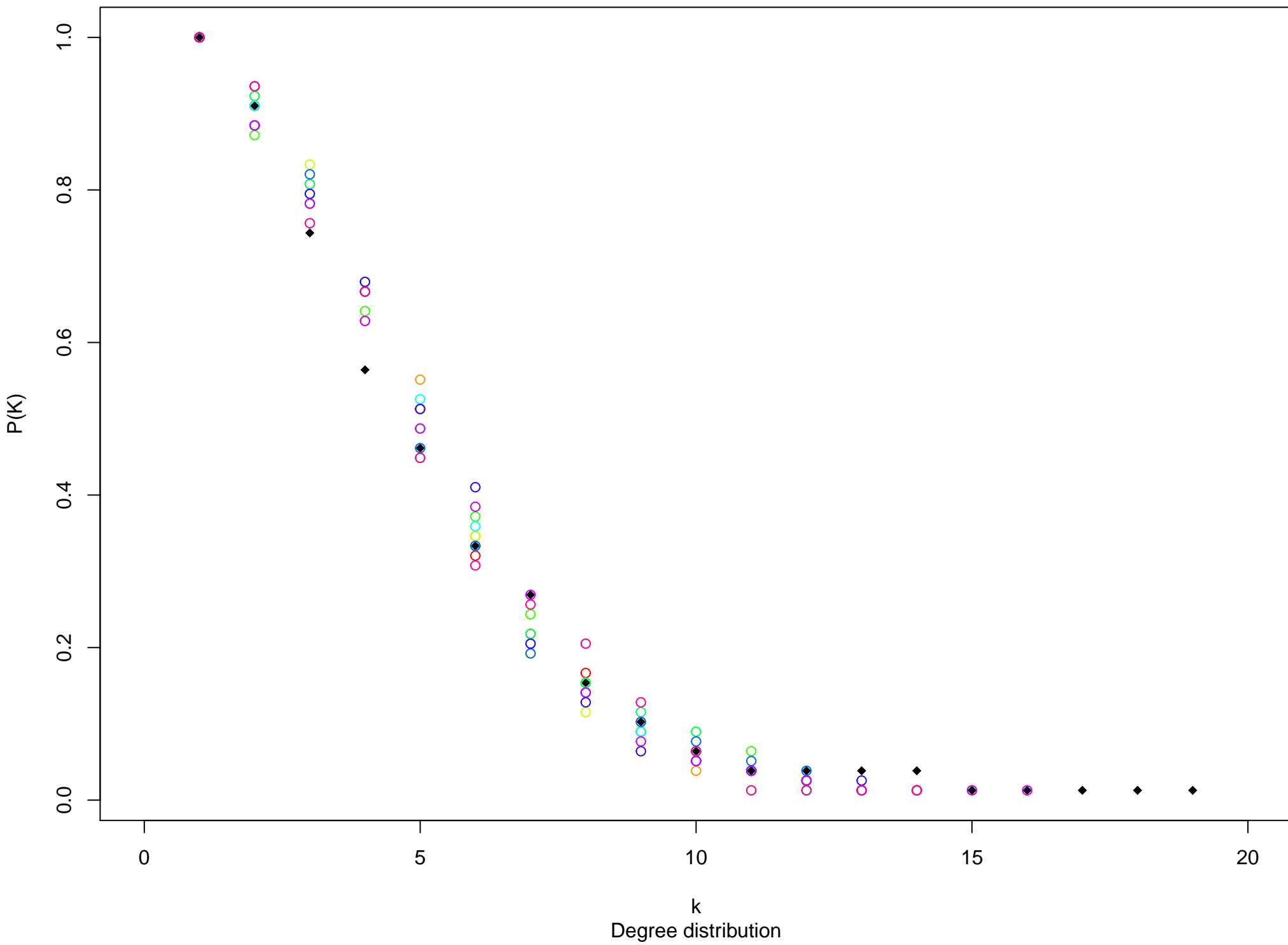

Supplement: Supplementary file 11 — Degree distribution for the disease to treatment (DTT) network (solid, black) and corresponding null model distributions (colored outlines); PDF format. (PDF 8 kb) [file 12918_2018_674_MOESM11_ESM.pdf]

# Treatment to Health

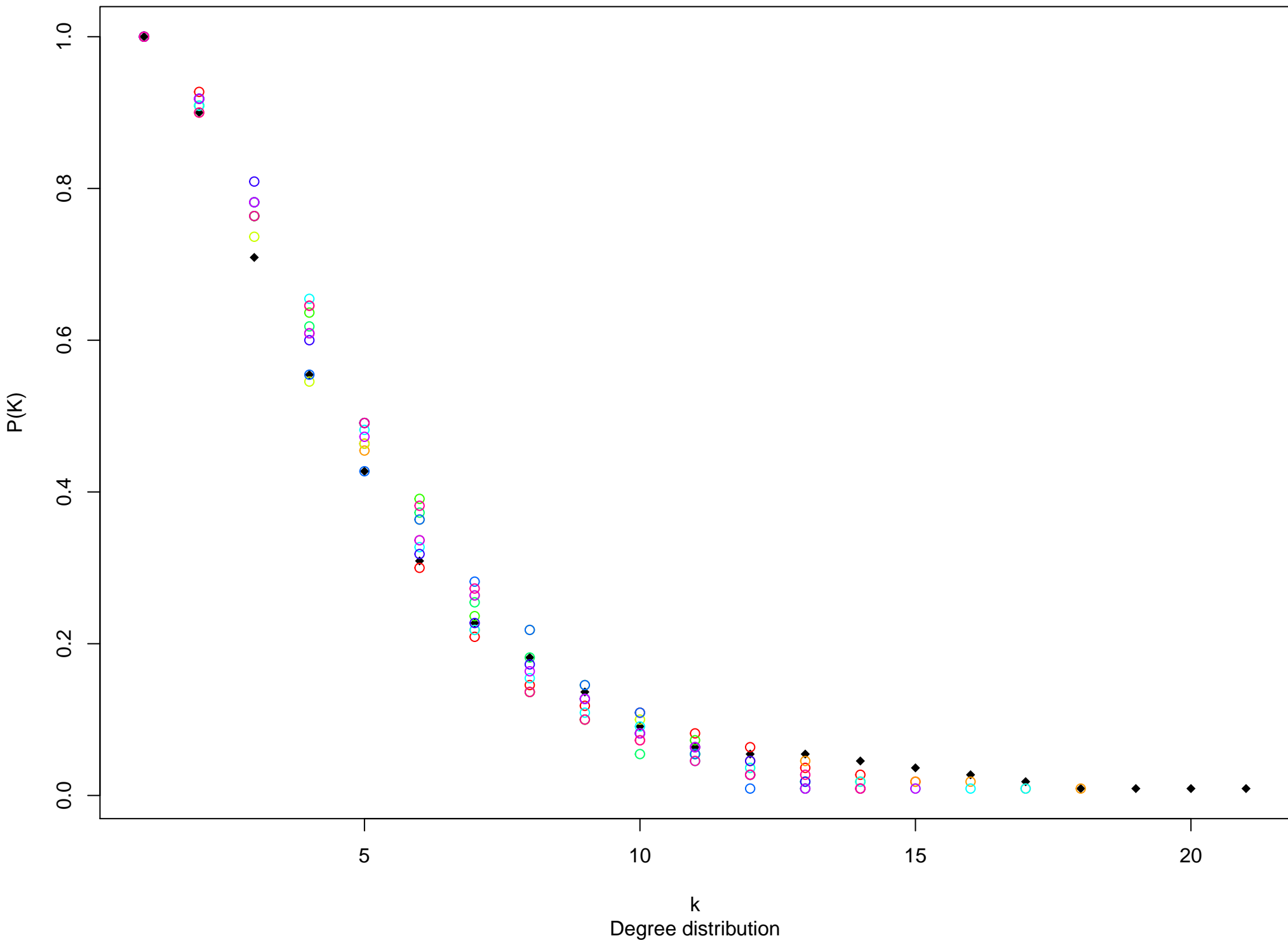

Supplement: Supplementary file 12 — Degree distribution for the treatment to health (TTH) network (solid, black) and corresponding null model distributions (colored outlines); PDF format. (PDF 9 kb) [file 12918_2018_674_MOESM12_ESM.pdf]
